# Characterization of a Read-through Fusion Transcript, BCL2L2-PABPN1, Involved in Porcine Adipogenesis

**DOI:** 10.3390/genes13030445

**Published:** 2022-02-28

**Authors:** Jiyuan Zhu, Zewei Yang, Wanjun Hao, Jiaxin Li, Liang Wang, Jiqiao Xia, Dongjie Zhang, Di Liu, Xiuqin Yang

**Affiliations:** 1 College of Animal Science and Technology, Northeast Agricultural University, Harbin 150030, China; zhujiyuan2008@163.com (J.Z.); yangzewei1997@163.com (Z.Y.); haowanjun1109@163.com (W.H.); ljxneau@163.com (J.L.); xiajiqiao365@163.com (J.X.); 2 Institute of Animal Husbandry, Heilongjiang Academy of Agricultural Sciences, Harbin 150086, China; wlwl448@163.com (L.W.); djzhang8109@163.com (D.Z.)

**Keywords:** adipogenesis, BCL2L2-PABPN1, chimeric RNA, *cis*-SAGe, genome-wide analysis, RNA-Seq

## Abstract

*cis*-Splicing of adjacent genes (*cis*-SAGe) has been involved in multiple physiological and pathological processes in humans. However, to the best of our knowledge, there is no report of *cis*-SAGe in adipogenic regulation. In this study, a *cis*-SAGe product, BCL2L2–PABPN1 (BP), was characterized in fat tissue of pigs with RT-PCR and RACE method. BP is an in-frame fusion product composed of 333 aa and all the functional domains of both parents. BP is highly conserved among species and rich in splicing variants. BP was found to promote proliferation and inhibit differentiation of primary porcine preadipocytes. A total of 3074/44 differentially expressed mRNAs (DEmRs)/known miRNAs (DEmiRs) were identified in porcine preadipocytes overexpressing BP through RNA-Seq analysis. Both DEmRs and target genes of DEmiRs were involved in various fat-related pathways with MAPK and PI3K-Akt being the top enriched. *PPP2CB*, *EGFR*, *Wnt5A* and *EHHADH* were hub genes among the fat-related pathways identified. Moreover, ssc-miR-339-3p was found to be critical for BP regulating adipogenesis through integrated analysis of mRNA and miRNA data. The results highlight the role of *cis*-SAGe in adipogenesis and contribute to further revealing the mechanisms underlying fat deposition, which will be conductive to human obesity control.

## 1. Introduction

Fat is a major factor affecting pig growth, development, and meat quality. Intramuscular fat (IMF) content is determinant of pork marbling and closely related to the juiciness, flavor and tenderness of pork. A suitable IMF content can bring a better taste and is important in improving pork quality [1]. However, back-fat thickness is negative related to lean meat yield [2]. The regulation of fat content and distribution in the body will bring major economic benefits to pig producers, which need to understand the mechanisms underlying fat deposition. Additionally, as an important endocrine organ, fat tissue plays key roles in maintaining body energy balance and glucose homeostasis [3], and is directly associated with some metabolic diseases, including diabetes and obesity. Pigs are similar to human beings in anatomy and physiology and have long been used as models in biomedical research [4,5,6]. Studies on adipogenesis in pigs will contribute to controlling metabolic diseases associated with fat.

It has been made clear that various transcription factors [7,8,9], signal transduction pathways [10,11,12], epigenetic factors [13,14], and functional RNAs [15,16] are involved in adipogenesis. However, adipogenesis is a complicated and precisely orchestrated process, and there are still many factors remaining to be identified before fully revealing the molecular mechanisms underlying adipogenesis. Chimeric RNA molecules are composed of exons from two independent genes. They can be produced by several mechanisms, including chromosome rearrangement, *cis*-splicing of adjacent genes (*cis*-SAGe), and *trans*-splicing [17]. *cis*-SAGe is cotranscription of adjacent genes coupled with intergenic splicing and forms read-through fusion transcripts [18,19]. Chimeric RNAs were first identified in tumor cells and once considered unique to tumors, which has focused researchers on their roles in carcinogenesis. They are involved in various tumors, and in some cases, can be used as diagnosis markers [20,21,22]. As research has progressed, chimeric RNAs have been found in normal tissues and can produce many fusion proteins, increasing greatly the complexity and diversity of the proteome. They can regulate gene expression, cell growth, vitality, and motility in normal physiological processes [23,24]. For example, PAX3–FOXO1 is needed for muscle lineage commitment [25,26], and DUS4L–BCAP29 is involved in neuronal differentiation [27].

The existing findings highlight the vital importance of chimeric RNAs and more researchers are paying attention to them. However, there are no studies on chimeric RNAs in adipogenesis in mammals. Here, we first identified a chimeric RNA produced by *cis*-SAGe, BCL2L2–PABPN1 (BP), in pigs and elucidated that it inhibited adipogenesis through MAPK and PI3K-Akt signaling pathways. The results highlight the role of read-through fusion transcripts in adipogenesis and contribute to further revealing the mechanisms underlying fat formation.

## 2. Materials and Methods

### 2.1. Animals, Nucleic Acid Isolation and cDNA Synthesis

All pigs were from the Institute of Animal Husbandry, Heilongjiang Academy of Agricultural Sciences (Harbin, China). The animal study was reviewed and approved by the Animal Care Committee of Northeast Agricultural University (Harbin, China). Fat tissues were obtained from 6-month-old Min and Yorkshire pigs raised in the same condition or from newborn Min pigs. RNA was extracted with TRIzol reagent (Invitrogen, Carlsbad, CA, USA) and reverse transcribed into cDNA with PrimeScriptTM 1st Strand cDNA Synthesis Kit (Takara, Dalian, China). In analysis of chimera formation, the reverse transcription (RT) primer was random 6 mers provided by the kit. 

### 2.2. Hematoxylin and Eosin Staining and Triglyceride Assay

Adipose tissues were fixed in 4% paraformaldehyde solution, dehydrated in ethanol, and embedded in paraffin. 4 μm thickness section was sliced with HistoCore BIOCUT (Leica, Nussloch, Germany) and stained with hematoxylin and eosin (HE) for morphological analysis. Tissues from three pigs in each breed were used and more than five fields were chosen for morphological analysis. Adipocyte size was determined with Leica Application Suite V4 (Leica). Triglyceride (TG) contents were measured with an enzymatic TG assay kit (GPO-POD; Applygen, Beijing, China) according to the manufacturer’s protocol.

### 2.3. Chimeric RNA Identification and cDNA Cloning

We previously obtained high-throughput paired-end RNA-seq data of fat tissues from Min and Yorkshire pigs [28], from which chimeric RNA was identified using ChimeraScan program [29] with the reference genome (*S. scrofa* 10.2) [30] using default parameters. Only read-through fusion candidates covering neighboring genes on the same strand of DNA were considered in this study. Other chimera candidates including inter-, intra-chromosomal, and adjacent ones were discarded.

Reverse transcription-polymerase chain reaction (RT-PCR) was used for validation of candidate BCL2L2-PABPN1 (BP), produced by B-cell lymphoma 2-like 2 protein (*BCL2L2*) and poly(A) binding protein nuclear 1 (*PABPN1*) genes, with specific primer pair, B1F/P1R, designed according to result of bioinformatic analysis and cDNA template from fat tissues. Another primer P2R designed according to porcine *PABPN1* mRNA (GenBank No. NM_001243548) was used to extend the chimera with B1F. The 5′ rapid amplification of cDNA ends (RACE) was used to clone the 5′ sequence using specific primers P1R and BPR, which is complementary to the junction of chimera with SMARTer RACE 5′/3′ kit (Takara). Primer P3F was used in the 3′ RACE reaction together with B1F.

Additionally, the 5′ RACE method was used to replenish the sequence of porcine *PABPN1* mRNA in which the outer and inner primers were P1R and P3R, respectively. P3R was complementary to exon 1 of porcine PABPN1. RT-PCR was performed with forward primer, P4F, complementary to the 5′ untranslated region (UTR) obtained and reverse primer, P4R, complementary to 3′ end of the chimera to verify the cDNA sequence of porcine *PABPN1*. Genomic structure was analyzed with BLAT program in UCSC genome (http://genome.ucsc.edu/, accessed on 9 March 2021). All primer sequences used in this study are listed in Table S1-1.

### 2.4. Primary Preadipocyte Isolation and Culture

Subcutaneous fat tissues were obtained after the newborn Min pigs were slaughtered, washed with sterile phosphate-buffered saline (PBS), and potentially contaminated muscle and connective tissue was carefully removed. After washing three times in PBS containing 1% penicillin–streptomycin (Invitrogen), fat tissues were cut into small pieces and digested with 0.1% type I collagenase (Invitrogen) for 40–50 min at 37 °C, then mixed with equal volumes of culture medium supplemented with penicillin–streptomycin and 10% fetal bovine serum (FBS) (Sigma, St. Louis, MO, USA), and filtered through 400-mesh filters. The filtrates were centrifuged at 1000 rpm for 5 min. The cell precipitation was resuspended with Dulbecco’s modified Eagle’s medium/Nutrient Mixture F-12 (DMEM/F12) containing 10% FBS and 1% penicillin–streptomycin. The medium was changed every 2 days until cells were grown to a desired density.

### 2.5. Preadipocyte Differentiation and Oil Red O Staining

To induce differentiation, DMEM/F12 was supplemented with 10% FBS, 0.5 mmol/L 3-isobytyl-1-methylxanthine, 1 μmol/L dexamethasone and 5 μg/mL insulin in which cells were incubated for 2 days. The cells were cultured with DMEM/F12 containing 10% FBS and 5 μg/mL insulin to maintain their differentiation until further analysis. The medium was changed every 2 days.

The differentiated adipocytes were stained with Oil Red O kit (Leagene, Beijing, China). The stained lipid droplets were viewed under a light microscope and photographed (Carl Zeiss AG, Jena, Germany). For quantification analysis, cellular Oil Red O was extracted with isopropanol and measured with optical absorbance at 510 nm.

To evaluate effects of BP on preadipocyte differentiation, overexpression vector of BP (pCMV-HA-BP) was constructed with pCMV-HA backbone at sites of *Eco*R I and *Kpn* I and transiently transfected with Lipofectamine 2000 (Invitrogen) according to manufacturer’s protocol. At 24 h after transfection, cells were subjected to differentiation inducement.

### 2.6. Real-time Quantitative PCR

Real-time quantitative PCR (qPCR) was performed with TB Green^®^ Premix Ex Taq^TM^ reagent kit (Takara). The PCR volume and reaction program were set strictly according to the manufacturer’s instructions. β-Actin was used as a reference and the relative expression level was analyzed with the 2^−ΔΔCt^ method [31].

### 2.7. Cell Counting Kit-8 Assay

Porcine preadipocytes were transiently transfected with pCMV-HA-BP or empty vector pCMV-HA for 24 h and further cultured until the CCK-8 assay was performed. In CCK-8 assays, cells were incubated with 10% CCK-8 (Beyotime, Shanghai, China) in complete medium for 2 h at 37 °C. The absorbance of cells was measured at 450 nm using a Tecan Microplate Reader Infinite F50 (Tecan GENios, Mannendorf, Switzerland).

### 2.8. Flow Cytometry

Porcine preadipocytes were inoculated in six-well plates at a density of 1 × 10^6^ cells per well and cultured for 24 h. Cells were transfected with pCMV-HA-BP or empty vector and cultured for another 24 h. After digested with trypsin, cells were washed with PBS, and stained with cell cycle staining Kit (MultiSciences, Hangzhou, China). Then the cell cycle was analyzed with FACSCalibur Flow Cytometer (Becton Dickinson, Franklin Lakes, NJ, USA).

### 2.9. Illumina-Seq Library Construction and Sequencing

The recombinant adenoviruses were constructed using the AdEasy system (Hanbio, Shanghai, China) as described by He and colleagues (1998). BP CDS were inserted into the shuttle plasmid containing an enhanced green fluorescent protein (EGFP) and cytomegalovirus promoters using *Kpn* I and *Xho* I sites, and homologous recombination was performed in *Escherichia coli* BJ5183 with adenoviral backbone pAdEasy 1. The recombinant adenovirus plasmid was packaged in HEK-293A cells after linearized with *Pac* I. Preadipocytes were infected with adenovirus virions at multiplicity of infection (MOI) of 300. At 48 h post-infection, cells were collected for RNA-Seq with Illumina NovoSeq 6000 platform (Illumina, San Diego, CA, USA) by Geneseeq Technology (Nanjing, China) using pair-end sequencing strategy according to the manufacturer’s protocols. Cells treated with empty adenovirus were used as a control. A total of six Ribo-Zero RNA-sequencing libraries including overexpressing and control groups were constructed, each with three replicates.

### 2.10. Genome-Wide mRNA Analysis

The raw reads were processed as described elsewhere [32]. High quality reads were mapped to reference genome of *S. scrofa* (11.1) using HISAT2 program [33]. Transcript abundances were quantified with StringTie software [34], and normalized with FPKM (Fragments per kilobase of transcript per million mapped reads) method across libraries. DESeq2 [35] was used to identify differentially expressed mRNAs (DEmRs) with an absolute log_2_-fold change ≥ 1 and *p* < 0.05. To functionally annotate DEmRs, Gene Ontology (GO) analysis was performed with Blast2GO with a cutoff E-value of 10^−5^; Kyoto Encyclopedia of Genes and Genomes (KEGG) [36] pathway analysis was done using KEGG Orthology Based Annotation System (KOBAS 3.0) [37] with default parameters. Protein-protein interaction (PPI) network was constructed with STRING database (https://string-db.org, accessed on 3 August 2021) and visualized with Cytoscape software (version 3.8.2).

### 2.11. Genome-Wide miRNA Analysis

The raw reads were filtered as described elsewhere [32]. Briefly, clean reads were first obtained, and then non-coding RNA (rRNA, tRNA, scRNA, snRNA, snoRNA, etc.) and those reads aligned to exon, intron, and repeat sequences were removed. The remained clean reads were searched against miRbase database (Release 22.1) to characterize known miRNAs in *S. scrofa*. Furthermore, novel miRNAs were predicted with MiRDeep2 [38]. The miRNA expression level was normalized to transcripts per million (TPM), and the DESeq2 software [35] were used to characterize differentially expressed miRNA (DEmiRs) with absolute log_2_-fold change ≥ 1 and *p* < 0.05. Target genes were predicted with miRanda program [39,40].

### 2.12. Statistical Analysis

All experiments were performed at least three independent times, each with three repeats. Representative data of one experiment were given as mean ± standard error (SE). Data were analyzed with SPSS19.0 software (SPSS; Chicago, IL, USA) and Student’s *t*-test was used to analyze differences between two groups. *p* < 0.05 (indicated with *) was considered statistically significant, and *p* < 0.01 (**) was considered very significant.

## 3. Results

### 3.1. Identification of Read-through Chimeric RNA Associated with Fat Content

**Figure 1:**
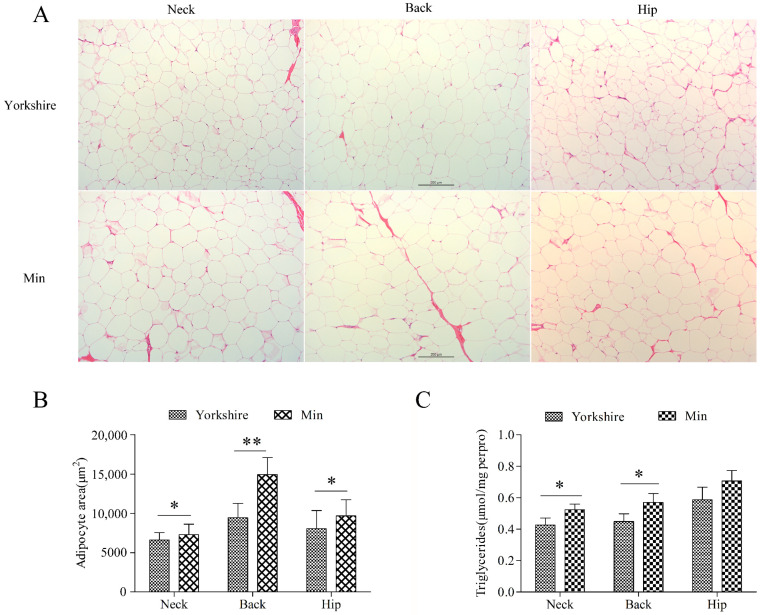
Comparison of subcutaneous adipose tissues between Yorkshire and Min pigs. (**A**) Morphological analysis with HE staining. (**B**) Adipocyte area of subcutaneous adipose tissues. The bar is 200 μm. (**C**) Triglyceride contents in subcutaneous adipose tissues. The data are shown as mean ± standard error. * and ** indicate *p* < 0.05, and *p* < 0.01, respectively.

Three subcutaneous fat tissues from neck, back, and hip were analyzed. Morphological analysis showed no difference between these tissues of Min and Yorkshire pigs (Figure 1A). There is significant difference in the average adipocyte area (*p < 0.05*) and TG content (*p < 0.05*) in subcutaneous tissues between Yorkshire and Min pigs except for TG content in hip fat, indicating that the subcutaneous fat deposition is different between Min and Yorkshire pigs (Figure 1B,C). Thus, backfat tissues from Min and Yorkshire pigs were used to screen chimeric RNAs related to fat deposition.

**Figure 2:**
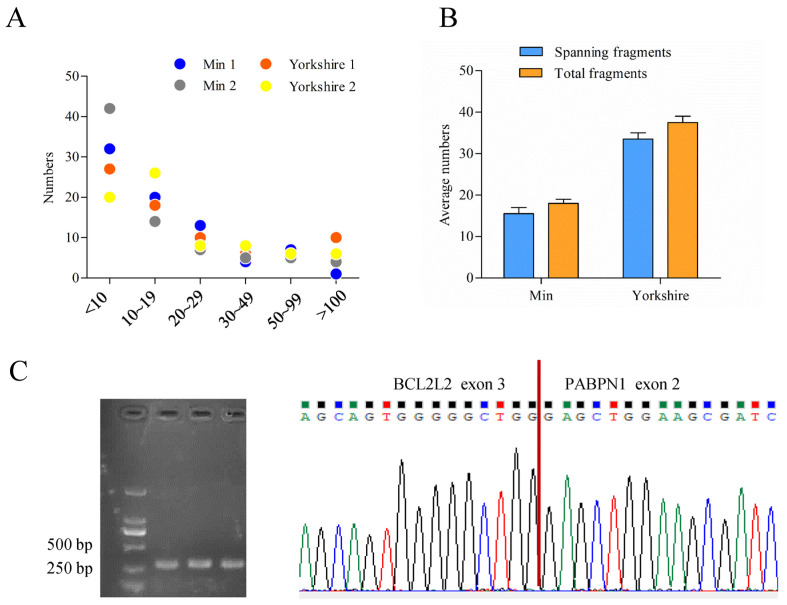
Characterization of porcine BCL2L2-PABPN1 (BP). (**A**) Score distribution of read-throughs identified. (**B**) Fragment distribution of BP in Min and Yorkshire pigs determined by ChimeraScan. (**C**) Confirmation of BP with RT-PCR and sequencing.

The bioinformatic analysis of data obtained previously [28] called 77 read-throughs that presented in all of the four samples by ChimeraScan (Table S2). Most of them had low score values which mean total fragments supporting chimera, indicating humble expression levels of read-throughs (Figure 2A). Among the read-throughs identified, one tag named BP presented with differential score between fat tissues of Min and Yorkshire pigs. Furthermore, fragments spanning breakpoint junction accounted for a very large proportion in the total fragments of BP (Figure 2B). Through RT-PCR and sequencing analysis, the existence of BP was confirmed (Figure 2C).

### 3.2. cDNA Cloning of BCL2L2–PABPN1

RACE analysis showed that porcine BP cDNA (V1) was 2097 bp in length and contained a complete CDS of 1002 bp, a 5′ UTR of 205 bp and a 3′ UTR of 890 bp. There was a typical poly(A) signal, AATAAA, at the 3′ end. It was located on chromosome 7 and composed of nine exons as revealed by BLAT program. The CDS spanned exons 3–9 with the first two exons and 8 bp of exon 3 comprising the 5′ UTR.

**Figure 3:**
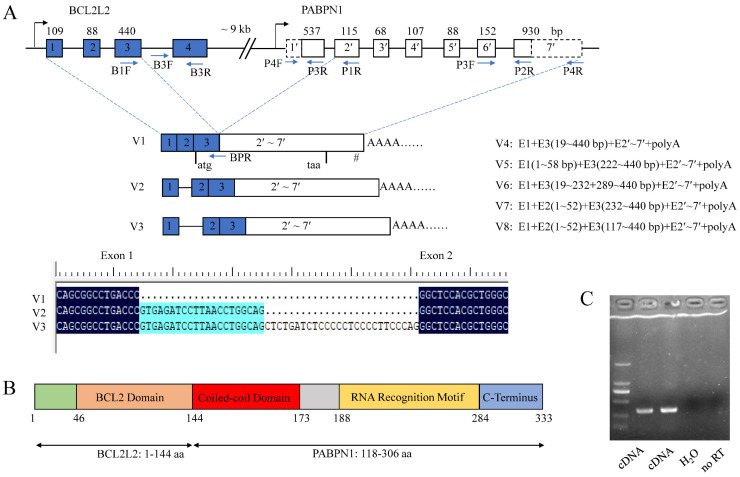
Characterization of porcine BCL2L2-PABPN1 (BP). (**A**) Genomic and mRNA structure of porcine BP. Boxes and lines indicate exons and introns, respectively. Dotted boxes in PABPN1 indicate that the sequences are first obtained here. Figures over the boxes indicate the length of the corresponding exons, while those in the boxes indicate exon No. Primer locations are shown with arrows. E, exon. I, intron. # indicates the position of poly(A) signal. Start and stop codons are shown under the boxes. (**B**) Motifs in the polypeptide of BP. (**C**) Identification of mechanisms underlying BP formation with RT-PCR. Templates were showed below. No RT, no reverse transcriptase control.

Seven alternative splicing (AS) variants of BP, named V2–V8, were obtained using 5′ RACE methods. V2 and V3 were formed by alternative 5′ splice sites (SSs) of exon 1, resulting in partial sequences of intron 1 being retained and having the same CDS as isoform V1. V4–V8 were absent of the start codon owing to the use of alternative 3′ SSs of exon 3 and thus could not be translated into a polypeptide. There were abundant alternative SSs in the first three exons of BP (Figure 3A). The sequences were deposited in GenBank under accession Nos. MH795109 for V1, and MW654158-64 for V2–V8.

Additionally, there was only an CDS sequence of porcine *PABPN1* deposited in GenBank (No. NM_001243548) with a small 3′ UTR of 63 bp, 5′ UTR of 8 bp, and absence of poly(A) signal (Figure 3A). Through cloning the cDNA of BP, a fusion product of *BCL2L2* and *PABPN1* genes, we have obtained the complete 3′ sequence of porcine *PABPN1* mRNA. Using 5′ RACE, the 5′ UTR of *PABPN1* was obtained and subsequent RT-PCR with primers complementary to 5′ UTR of *PABPN1* and the end of the last exon of BP, respectively, confirmed the sequence of *PABPN1*.

The *PABPN1* cDNA was 1997 bp in length with 186 bp of 5′ UTR, 921 bp of CDS encoding a polypeptide of 306 aa, and 890 bp of 3′ UTR (GenBank accession No. MH795126). The porcine PABPN1 protein was completely identical to that in humans (NM004643), and thus contained three major domains as its counterpart in humans [41]: an acidic N-terminal domain containing a stretch of 10 alanines and a coiled-coiled domain (CCD, spanning 119–146 aa), a single ribonucleoprotein-type RNA recognition motif (RRM, 161–257 aa), and a basic arginine-rich C-terminal region (258–306 aa).

Porcine BP is an in-frame fusion product with a molecular weight of 37.2 kDa and a pI of 8.58. The predicted polypeptide was composed of 333 aa with the first 144 aa from BCL2L2 (1–144 aa of BCL2L2) and the last 189 aa from PABPN1 (118–306 aa of PABPN1). Porcine BCL2L2 was composed of 193 aa and contained a functional BCL2 domain in the 46–144 aa as revealed by Blastn program. Thus, BP has all functional domains of both parents except for a stretch of 10 alanines in the N-terminal region of PABPN1 (Figure 3B).

### 3.3. Mechanisms Underlying BCL2L2–PABPN1 Formation

BP comprised nine exons with exons 1–3 from the first three exons of *BCL2L2* and 4–9 from the last six exons of *PABPN1*. BLAT analysis showed that *BCL2L2* and *PABPN1* were composed of four and seven exons, respectively. The configuration of BP, involving the second-to-last exon in the former gene joining to the second exon in the latter gene, was the most common type of *cis*-SAGe [42,43,44]. Additionally, *BCL2L2* was adjacent to *PABPN1* on porcine chromosome 7 with a distance smaller than 10 kb (~9 kb) and had the same transcription direction, which is another characteristic of the formation of *cis*-SAGe [45]. These make BP a candidate for *cis*-SAGe.

To confirm its transcriptional read-through nature, RT-PCR was used to detect primary mRNA as described by Qin and coworkers [43] in which RT primer (P1R) was annealed to the second exon of *PABPN1*, and PCR primers (B3F/R) were complementary to the last intron and the last exon of *BCL2L2*, respectively (Figure 3A). To avoid DNA contamination, total RNA was digested with DNase I and no reverse transcriptase control was used. The fragment was successfully amplified (Figure 3C), indicating that the transcript ran from *BCL2L2* to *PABPN1* and BP was a product of *cis*-SAGe.

There is a read-through product (NM_001199864) of *BCL2L2* and *PABPN1* in humans. Both the human and pig sequences had very high identities in aa, CDS, even in the complete cDNA. Additionally, there were also homologs in various orders of Mammalia including Primates, Cetacea, Even-toed ungulates, Chiropter, Carnivora, and even in *Ornithorhynchus anatinus*, one of the oldest mammals. Except for in humans, no sequences were described as read-through origin, but the identities were more than 95% between the polypeptide of porcine BP and any of the homologs (Table S1-2). These indicated that BP was highly conserved in evolution and might have critical roles in life.

### 3.4. Effects of BCL2L2–PABPN1 on Preadipocyte Proliferation and Differentiation

**Figure 4:**
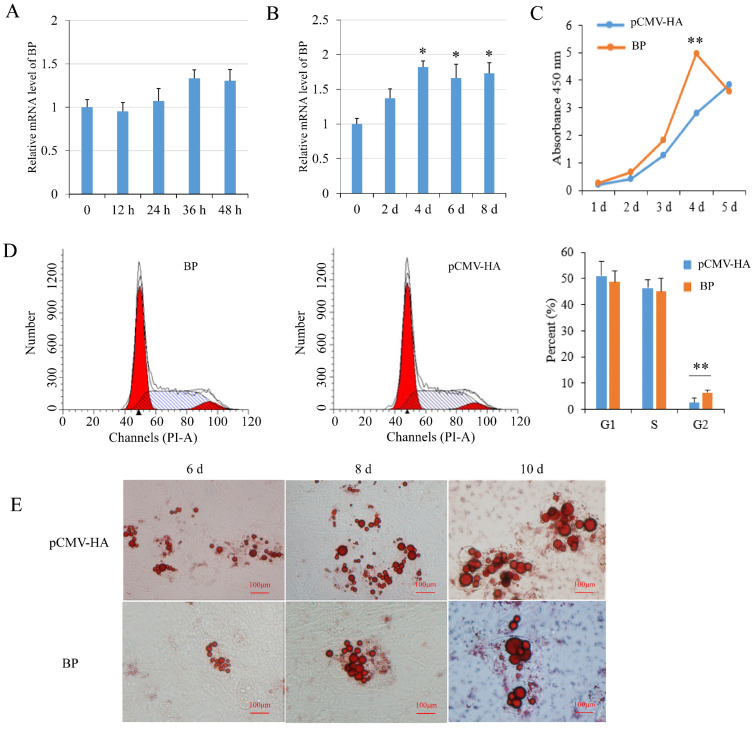
Effects of BCL2L2-PABPN1 (BP) on subcutaneous preadipocyte proliferation and differentiation. (**A**) Expression of BP during porcine preadipocyte proliferation determined with real-time PCR. (**B**) Expression of BP during porcine preadipocyte differentiation determined with real-time PCR. (**C**) Effect of BP on cell proliferation measured by CCK-8 assay. (**D**) Effects of BP on cell cycle measured with flow cytometry. (**E**) Oil red O staining of differentiated adipocyte. * and ** indicates *p* < 0.05 and *p* < 0.01, respectively, compared with control groups. The bar is 100 μm.

BP mRNA level had a tendency to increase during preadipocyte proliferation but the changes were not significant (*p* > 0.05) (Figure 4A). During preadipocyte differentiation, the expression of BP was increased significantly (*p* < 0.05) since 4 days after induction compared with non-induced cells (Figure 4B). Real-time PCR and Western blot analysis showed that BP expression was increased effectively in preadipocytes transfected with the plasmids pCMV-HA-BP. CCK-8 assay showed that BP overexpression increased cell number compared to the cells transfected with empty vector, with the highest level (*p* < 0.01) at 4 days post-transfection (Figure 4C). Flow cytometry analysis showed that the number of G2-phase preadipocytes was increased significantly (*p* < 0.01) in groups overexpressing BP (Figure 4D). Overexpression of BP resulted in a decrease of lipid droplets compared to the control cells at 6-, 8- and 10-days post-induction as revealed with Oil Red O staining (Figure 4E). The results indicated that BP promoted proliferation and inhibited differentiation of preadipocytes.

### 3.5. Genome-Wide Identification of mRNAs Involved in BCL2L2-PABNP1 Regulation

**Figure 5:**
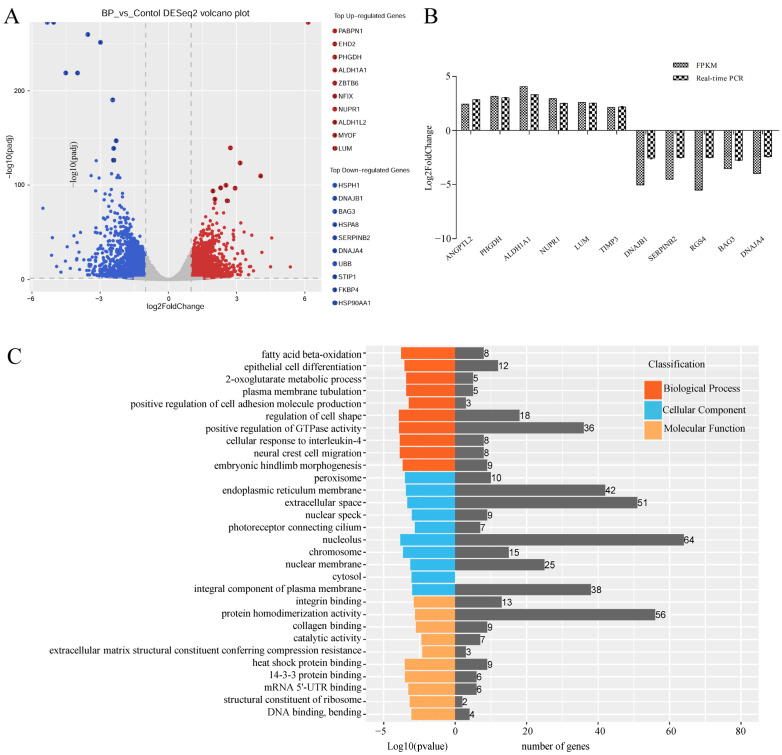

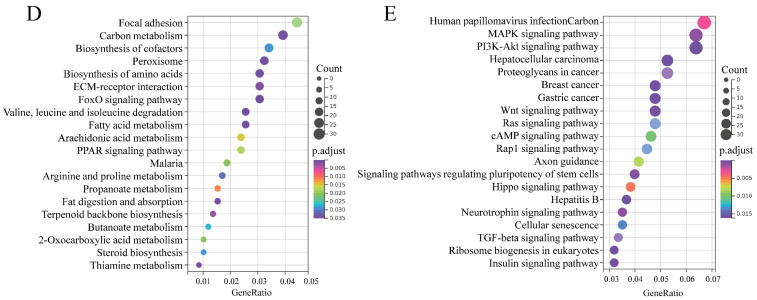
Characterization of differentially expressed mRNAs (DEmRs) induced by BCL2L2-PABPN1. (**A**) Volcano plot of DEmRs. (**B**) Validation of RNA-Seq data with real-time PCR method. (**C**) GO enrichment analysis of DEmRs. (**D**) KEGG pathway analysis of upregulated DEmRs. (**E**) KEGG pathway analysis of downregulated DEmRs.

RNA-Seq technology was used to explore mechanisms of BP on adipogenesis in cells transfected with adenoviruses expressing BP at a condition of MOI 300 and 48 h which was predetermined with fluorescence microscope and qPCR analysis (Figure S1). An average number of 104,791,770 and 128,873,418 raw reads were obtained in control and treatment groups, respectively. After removal of low-quality and adaptor containing reads, 103,766,967 and 127,695,699 clean reads were obtained. In these clean data, the Q30 content was more than 92.67%. A total of 3074 DEmRs were obtained by RNA-Seq analysis, among which 1476 were upregulated and 1598 were downregulated in cells overexpressing BP compared with control groups (Figure 5A, Table S3-1). Eleven DEmRs were selected randomly to validate RNA-Seq data with qPCR, and consistent results were obtained (Figure 5B).

To highlight the function of DEmRs, GO and KEGG analysis were performed. GO analysis revealed that the DEmRs were involved in multiple categories in molecular functions, cellular component, and biological processes (Figure 5C). The major biological processes enriched included fatty acid beta-oxidation, 2-oxoglutarate metabolic process, and 2-oxoglutarate metabolic process, etc. KEGG analysis performed on all DEmRs revealed that various fat-related pathways, such as MAPK, TGF-β, Wnt, PI3K-Akt, and Fatty acid metabolism, etc. were significantly enriched. When the up- and downregulated DEmRs were subjected to KEGG analysis separately, the pathways enriched suggested different roles between them. The upregulated DEmRs were mainly involved in fat metabolism-related pathways including fatty acid metabolism, fat digestion and absorption, arachidonic acid metabolism, butanoate metabolism, propanoate metabolism, and steroid biosynthesis; additionally, two signaling pathways associated with adipogenesis, PPAR and FoxO, were enriched by upregulated DEmRs. Downregulated DEmRs were enriched in some adipogenesis-related signaling pathways including MAPK, PI3K-Akt, Wnt, TGF-beta, insulin, Hippo, and cAMP signaling pathways, with MAPK and PI3K-Akt being the top two enriched pathways except for human papillomavirus infection whose enrichment might be associated with adenovirus infection (Figure 5D,E). These results indicated that DEmRs induced by BP overexpression were involved in fat deposition, which confirmed the role of BP in adipogenesis and highlighted the underlying mechanisms.

**Figure 6:**
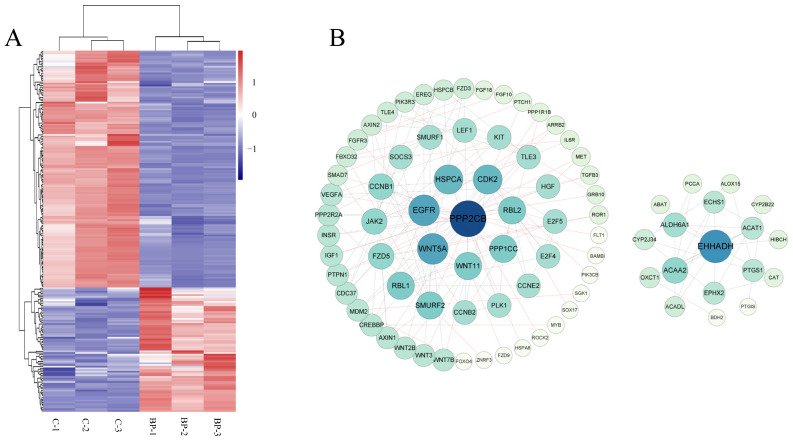
Characterization of differentially expressed mRNAs (DEmRs) involved in fat-related pathways. (**A**) Heatmap cluster of DEmRs involved in fat-related pathways. (**B**) The protein-protein interaction network of DEmRs involved in fat-related pathways. The size of the circle indicates the degree of interaction between the genes. The network was constructed with score > 0.9, FDR stringency = 1 percent, and disconnected nodes were hide.

The above 15 fat-related pathways involved 212 DEmRs of which 73 were upregulated and 139 were downregulated in response to BP treatment (Figure 6A, Table S3-2). PPI network analysis showed that these DEmRs were grouped into two separate modules. In module 1 the top three key nodes were PPP2CB, EGFR, and Wnt5A with a degree of 11, 9, and 9, respectively. Both PPP2CB and EGFR are involved in MAPK and PI3K-Akt signaling pathways. In module 2, EHHADH, ACAA 2, and ALDH6A1 were the top three key nodes with a degree of 10, 6, and 5, respectively (Figure 6B).

### 3.6. Genome-Wide Identification of miRNAs Involved in BCL2L2-PABNP1 Regulation

**Figure 7:**
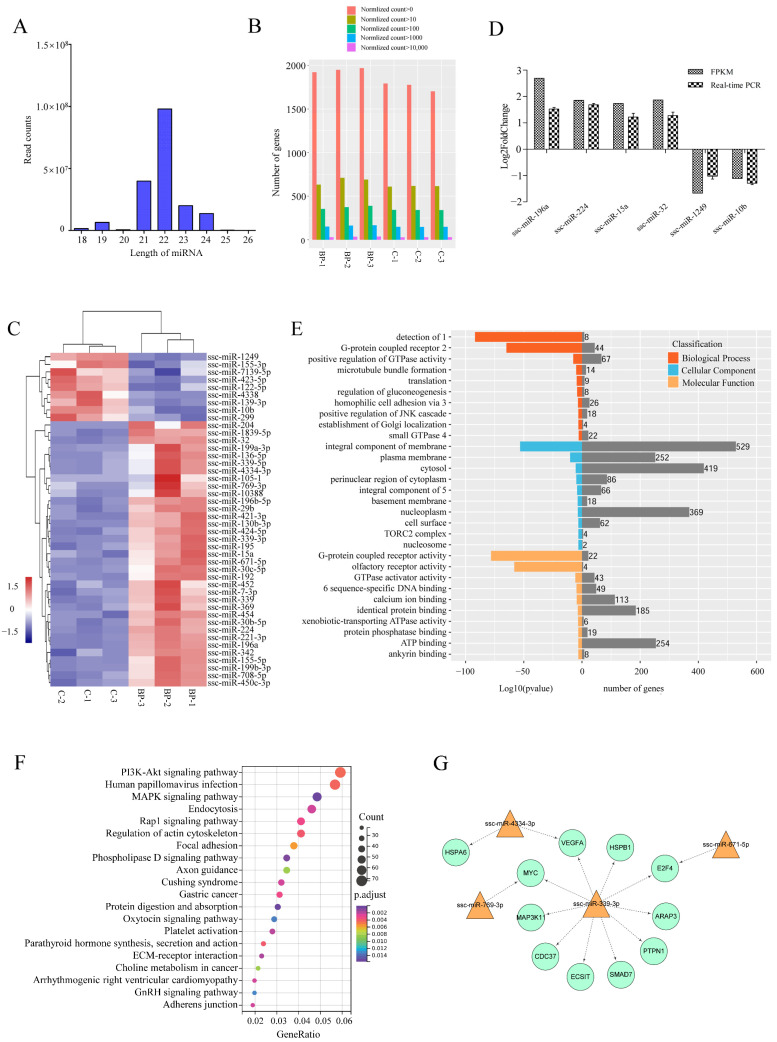
Characterization of differentially expressed miRNAs (DEmiRs) induced by BCL2L2-PABPN1. (**A**) Length distribution of miRNAs among six samples. (**B**) Barplot profiling the expression levels of the miRNAs in each sample. (**C**) Heatmap cluster of known DemiRs. (**D**) Validation of Illumina data with real-time PCR. (**E**) GO enrichment of target genes of DEmiRs. 1, detection of chemical stimulus involved in sensory perception of smell; 2, G-protein coupled receptor signaling pathway; 3, homophilic cell adhesion via plasma membrane adhesion molecules; 4, small GTPase mediated signal transduction; 5, integral component of plasma membrane; 6, RNA polymerase II core promoter proximal region sequence-specific DNA binding. (**F**) KEGG enrichment of target genes of DEmiRs. (**G**) Fat-related miRNA-mRNA interaction network.

To further characterize the mechanisms underlying the regulation of BP on adipogenesis, transcriptomic miRNA alteration induced by BP was analyzed using Illumina RNA sequencing. An average number of 25,583,601 and 18,115,912 clean reads (96.01% and 95.51% of raw reads) of which the average percentage of miRNAs was 63.72 and 65.80 were obtained from control and BP-treated groups, respectively. On the basis of *S. scrofa* genome (11.1), a total of 1987 unique miRNAs were identified (Table S4-1). The lengths of the miRNAs were mainly distributed in 19–24 nt in all six groups, with a maximum of 22 nt (Figure 7A). The expression level and count distribution of the total miRNAs in each sample was shown in Figure 7B. Compared with control groups, 44 known miRNAs were identified as differentially expressed miRNAs (DEmiRs) including 35 upregulated and nine downregulated during BP treatment (Figure 7C, Table S4-2). The expression of six DEmiRs were validated by real-time PCR, and consistent results were obtained between qPCR and RNA-Seq (Figure 7D).

A total of 4064 putative target mRNAs were predicted for these DEmiRs (Table S4-3), and top 10 target mRNAs of each DEmiRs were selected according to total score for GO and KEGG analysis. GO enrichment analysis showed that target genes were enriched in some categories of biological process such as positive regulation of GTPase activity, regulation of gluconeogenesis, and positive regulation of JNK cascade (Figure 7E). Two of the top three KEGG pathways enriched by target genes of DEmiRs were PI3K-Akt and MAPK signaling pathways both of which are important regulators of adipogenesis. Additionally, phospholipase D, Rap 1, and regulation of actin cytoskeleton signaling pathways were also significantly enriched (Figure 7F).

### 3.7. Integrated Analysis of mRNA and miRNA Data

To highlight DEmiRs involved in the regulation of BP on adipogenesis, integrated analysis was performed between known DEmiRs and 212 DEmRs involved in 15 fat-related pathways, in which those DEmRs identified as target genes of known DEmiRs and had a negatively correlated expression levels with the paired DEmiRs were selected for further analysis. A total of 11 differentially expressed target mRNAs (DETmRs) and four paired DEmiRs were obtained (Table S4-4). These fat-related DETmRs and the paired DEmiRs constituted a network in which ssc-miR-339-3p was critical (Figure 7G). Five of the 10 genes regulated by ssc-miR-339-3p, *MYC*, *VEGFA*, *MAP3K11*, *HSPB1* and *ECSIT*, are involved in MAPK signaling pathway.

## 4. Discussion

Chimeric RNAs were traditionally believed to be produced by chromosome rearrangement and unique to carcinogenesis until recent discoveries of RNA *trans*-splicing and *cis*-SAGe. It has been found that chimeric RNAs are expressed in noncancerous cells and tissues and involved in normal physiological process such as muscle lineage commitment [25,26] and neuronal differentiation [27]. However, there is no report on chimeric RNAs in fat formation in mammals.

Here, through analyzing our previous paired-end high-throughput sequencing data from backfat tissues of Min and Yorkshire pigs [28], chimeric RNA BP was characterized and the full-length cDNA was cloned in pigs using RT-PCR and RACE. Additionally, BP formation was identified as *cis*-SAGe. To the best of our knowledge, this is the first report on *cis*-SAGe in pigs. Min pig is a local breed in Northeast China with abundant fat content, while Yorkshire pigs have high lean meat percentage owing to long extensive breeding. BP was differentially characterized between fats from the two breeds. The role of BP in fat formation was thus expected and confirmed. The results extend the function of chimeric RNAs to adipogenesis, a normal physiological process that chimeric RNAs have not been involved in.

During cloning, we obtained seven AS variants of BP; some of which occurred in the 5′ UTR. The AS patterns identified here include exon skipping, alternative 5′ and 3′ SSs. It has been shown that exons closer to the intergenic region of two parental genes have lower conservation than those farther from the region and tend to be alternatively spliced in the formation of read-through chimeric RNA [44,46]. In the present study, AS variants were by-products in 5′ RACE cloning of BP and we did not focus on AS characterization. Additionally, the reverse primer of 5′ RACE was complementary to the junction of the two parents, resulting in inability to identify variants alternatively spliced in this region. Thereafter, there should be more AS variants remaining to be identified and more AS patterns might be present in BP. In a previous report analyzing formation and structures of *cis*-SAGe chimeric RNA [46], 20 and 23 transcript variants were obtained from ZNF343–SNPRB and COX17–POPDC2, respectively. These indicate that *cis*-SAGe is also rich in AS like regular pre-mRNAs.

As a nuclear poly(A) RNA binding protein, PABPN1 plays a key role in polyadenylation. It can directly interact with poly(A) polymerase (PAP) through the CCD domain leading to stimulation of the processivity of PAP [47], while binding to poly(A) RNA via the single RRM domain with a contribution from the C-terminal region [48]. BP contained all the functional domains of PABPN1 including CCD, RRM and C-terminal region implicated in polyadenylation, which provides a structural basis for polyadenylation. This suggests a role of BP in post-transcriptional regulation through mediating poly(A) tailing.

Because BP was identified in fat tissues, we focused on its role in adipogenesis and found that it can promote preadipocyte proliferation and inhibit differentiation. Mechanisms underlying the regulation of BP on adipogenesis were then analyzed with RNA-Seq, and genome-wide DEmRs and DEmiRs were characterized in preadipocytes overexpressing BP. Both DEmRs and target genes of DEmiRs were significantly involved in MAPK and PI3K-Akt, two of the important signaling pathways regulating adipogenesis. Various recent studies showed that PI3K-Akt pathway positively regulated the adipogenesis [49,50,51]. However, the role of MAPK signaling pathway in adipogenesis was bi-directional. Some reports demonstrated that activation of the MAPK pathway phosphorylates PPARγ, an adipogenic marker, and thus opposed adipogenesis [52,53,54]. While studies on effects of genes such as pigment epithelium-derived factor, a newly identified adipokine, miR-145 and lncRNA 332443 on adipogenesis revealed a positive role of MAPK signaling pathway during differentiation process [55,56]. These indicates that the role of MAPK in adipogenesis is complicated and versatile. Nevertheless, we showed that MAPK and PI3K-Akt were important for the regulation of BP on adipogenesis.

## 5. Conclusions

In this study, a read-through fusion transcript BP was first characterized in pigs. The deduced polypeptide contains the main functional domains of both parents, BCL2L2 and PABPN1, and highly conserved among species including *Ornithorhynchus anatinus*. BP was found to inhibit differentiation of primary porcine preadipocytes, and MAPK and PI3K-Akt were identified as the key signaling pathways affected by BP in which *PPP2CB* and *EGFR* were the hub genes. Additionally, ssc-miR-339-3p was critical for BP regulating adipogenesis. The results highlight the role of chimeric RNA in adipogenesis in mammals.

## Data Availability

All the relevant data are provided along with the manuscript as Supplementary Files. The RNA-Seq data associated with this study have been submitted to the NCBI SRA database (accession number GSE192412).

## Supplementary Materials

The following are available online at https://www.mdpi.com/article/10.3390/genes13030445/s1, Figure S1: Optimum condition for transfection of adenovirus, Table S1: Primers and identities of BP among species, Table S2: Read-throughs expressed in all of the four samples, Table S3: differentially expressed mRNAs in response to BP treatment, Table S4: miRNAs identified in this study.
