# Supplementary material for: Characterization of a Read-through Fusion Transcript, BCL2L2-PABPN1, Involved in Porcine Adipogenesis
Source: Genes (Basel). 2022 Feb 28;13(3):445. doi: 10.3390/genes13030445 (PMC8955228; doi:10.3390/genes13030445)
Supplement: Supplementary file 1 [file genes-13-00445-s001.zip › Figure S1 .pdf]

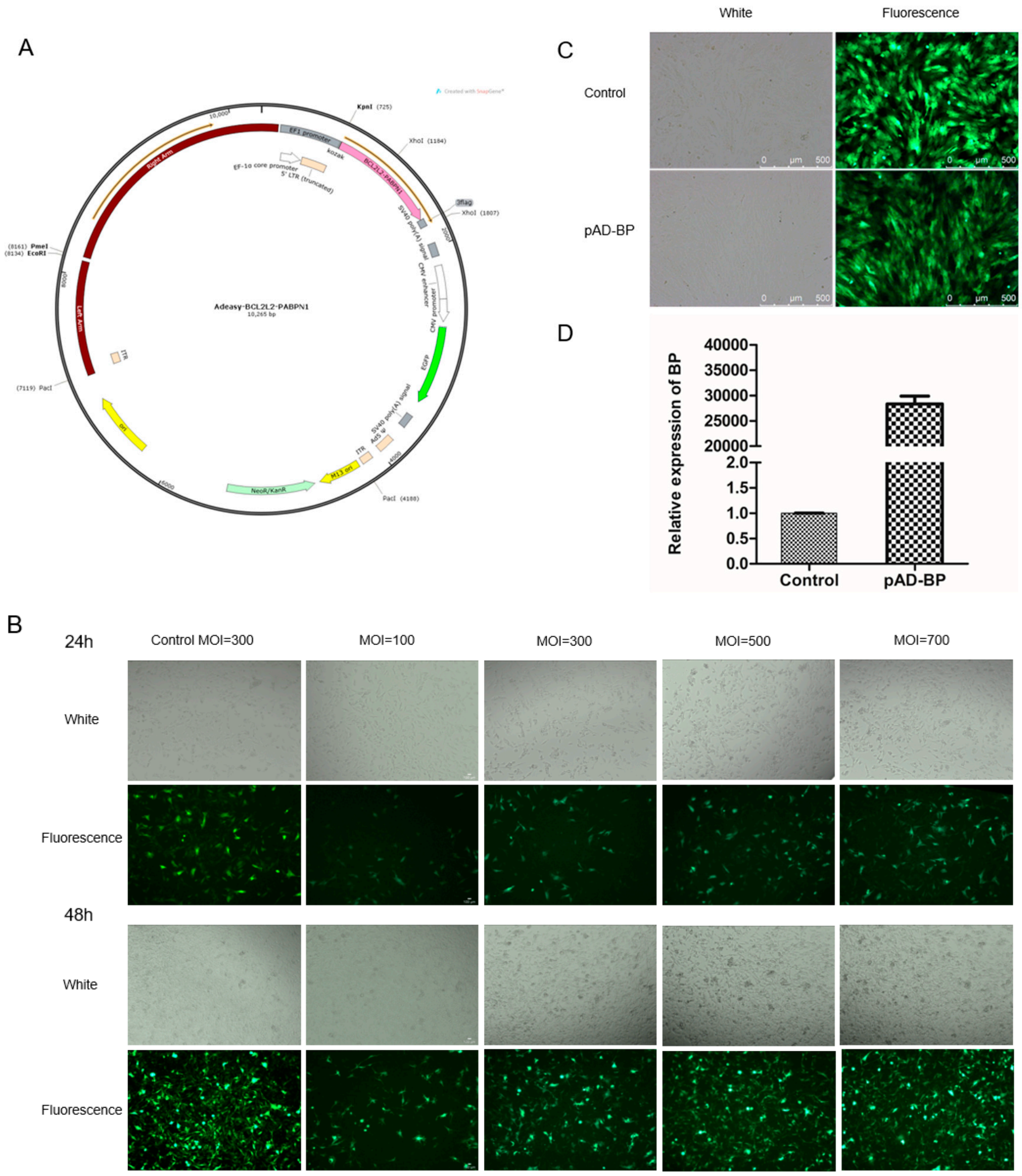

Figure S1. Optimum condition for transfection of adenovirus. (A) Diagram of adenovirus vector expressing porcine BCL2L2-PABPN1 gene (pAD-BP). (B) Screening of transfection condition for pAD-BP with fluorescence microscope. (C) Transfection of pAD-BP at MOI 300 and 48 h. (D) Real-time analysis of overexpression efficiency of pAD-BP. BP, BCL2L2-PABPN1. Cells transfected with empty adenovirus were used as control.
