# Supplementary material for: Characterization of a Read-through Fusion Transcript, BCL2L2-PABPN1, Involved in Porcine Adipogenesis
Source: Genes (Basel). 2022 Feb 28;13(3):445. doi: 10.3390/genes13030445 (PMC8955228; doi:10.3390/genes13030445)
Supplement: Supplementary file 1 [file genes-13-00445-s001.zip › label-read me.pdf]

Figure 2.C

Lane 1：DNA Ladders Marker

Lane 2-6：Confirmation of BP with RT-PCR

Figure 3.C

Lane 1：DNA Ladders Marker

Lane 2：cDNA（Identification of mechanisms underlying BP formation with RT-PCR）

Lane 3：cDNA（Identification of mechanisms underlying BP formation with RT-PCR）

Lane 4：H_2O

Lane 5：no RT
