# Supplementary material for: Characterization of a Read-through Fusion Transcript, BCL2L2-PABPN1, Involved in Porcine Adipogenesis
Source: Genes (Basel). 2022 Feb 28;13(3):445. doi: 10.3390/genes13030445 (PMC8955228; doi:10.3390/genes13030445)
Supplement: Supplementary file 1 [file genes-13-00445-s001.zip › Table S1.pdf]

Table S1-1 Primers used for cDNA cloning and verification of BCL2L2-PABPN1 formation

Name/Gene	Sequence (5'-3')
**cDNA cloning**	
B1	F: cttcacccaggtctctgatgaactc
P1	R: atattcatctgcttctctacttcgttc
P2	R: tctagcccggcccctgtagac
BP	R: tcgcttccagctcccagcccccactg
P3	F: aacagaccaggcatcagcacaacagac
P3	R: cctcagactccaagccgttcccgta
P4	F: ttgactaatgaatcctcggcg
P4	R: aaaatccacctcccacctcc
B3	F: tgcagtggatggaactggaac
B3	R: ttcctgctctcctgtccccc
**Tissue expression and real-time PCR analysis**	
BCL2L2-	F: ccaggtctctgatgaactc
PABPN1	R: ctgcttctctacttcgttc
β-actin	F: catcaccatcggcaacga
	R: gcgtagaggtccttcctgatgt
DNAJB1	F: ccccaaggaaggagaccaga
	R: aaagcctcccgaagggtaat
SERPINB2	F: tcctgtcggactgtacctc
	R: ctgggcagcacttgtattt
DNAJA4	F: cccatctacaaagcaccc
	R: catgtcgtccgttatcct
ANGPTL2	F: gctgacgaaccaaggcaact
	R: tggcatattcggcaaagacc
PHGDH	F: gaacgacagcacctttgcccttg
	R: gctccagcgcattgacccgact
ALDH1A1	F: atttaggaggctgcataaga
	R: aaagtttccatccatcggta
NUPR1	F: gctcctatgcccagaagatg
	R: ctccctcccaggtaagaatg
RGS4	F: catgctggagcccacgataa
	R: tttgaggaagcggcgatagg
LUM	F: tcccagatgagtatttcaagca
	R: gtattccactatcagccagttc
BAG3	F: tcccaggtcaagtccaggtctatg
	R: ttgctcttgtctgctgccatctc
TIMP3	F: ccgtgtctatgatggcaagatg
	R: caaggcaggtagtagcaggattt
**Stem-loop RT-PCR analysis**	
ssc-miR-1249	RT: gtcgtatccagtgcagggtccgaggtattcgcactggatacgactgaagaa

	F: acactccagctgggacgcccttcccccc
ssc-miR-196a	RT: gtcgtatccagtgcagggtccgaggtattcgcactggatacgaccccaacaa
	F: acactccagctgggtaggtagtttcatgt
ssc-miR-224	RT: gtcgtatccagtgcagggtccgaggtattcgcactggatacgactaaacgg
	F: acactccagctgggcaagtcactagtggt
ssc-miR-15a	RT: gtcgtatccagtgcagggtccgaggtattcgcactggatacgacacaaacc
	F: acactccagctgggtagcagcacataatg
ssc-miR-32	RT: gtcgtatccagtgcagggtccgaggtattcgcactggatacgacgcaactt
	F: acactccagctgggtattgcacattact
ssc-miR-10b	RT: gtcgtatccagtgcagggtccgaggtattcgcactggatacgacacaaatt
	F: acactccagctgggtaccctgtagaaccg
universal primer	R: cagtgcagggtccgaggtat
U6	RT and R: aacgcttcacgaatttgcgt
	F: ctcgcttcggcagcaca

F: forward, R: reverse, RT: reverse transcription

Table S1-2 Identities of procine BCL2L2-PABPN1 with orthologs from various species

Species	GenBank No.	Aa length	Identities with pig (%)	
			aa	cds
Human	NM_001199864	333	99.4	95.41
Alpaca	XM_031678858	333	99.4	96.41
Vulpe	XM_026007536	333	99.4	95.61
Whale	XM_024133866	333	99.4	96.61
Bat	XM_037129340	333	99.4	94.71
