# Supplementary figures and images for: Characterization of a Read-through Fusion Transcript, BCL2L2-PABPN1, Involved in Porcine Adipogenesis
Source: Genes (Basel). 2022 Feb 28;13(3):445. doi: 10.3390/genes13030445 (PMC8955228; doi:10.3390/genes13030445)

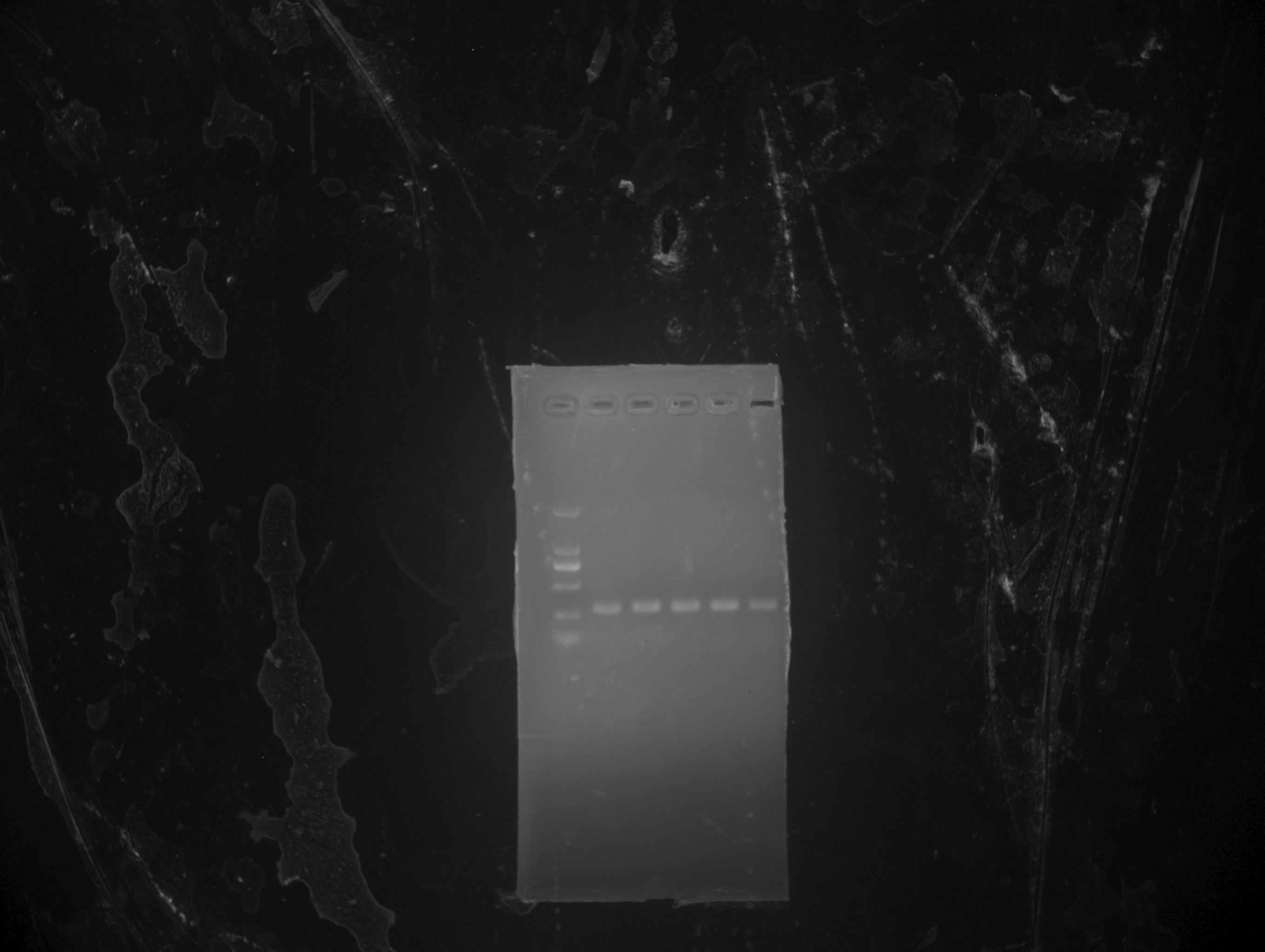

Supplement: Supplementary file 1 [file genes-13-00445-s001.zip › Figure 2.C-Lane 1ú║DNA Ladders Marker---Lane 2-6ú║Confirmation of BP.tif]

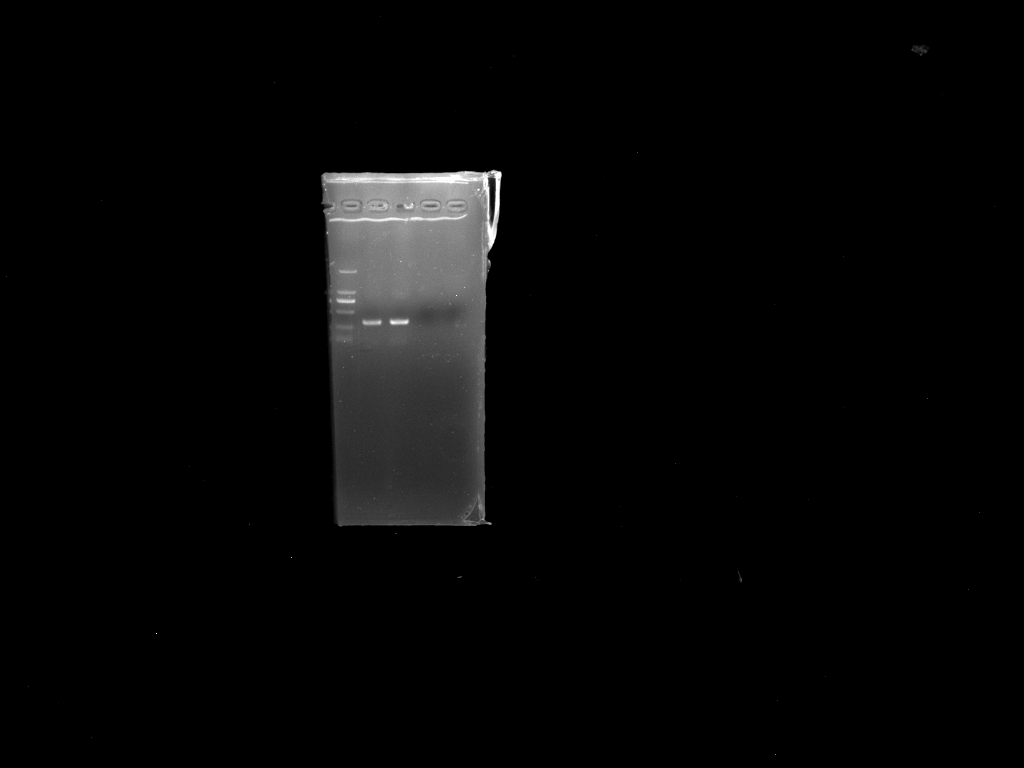

Supplement: Supplementary file 1 [file genes-13-00445-s001.zip › Figure 3.C-Lane 1ú║DNA Ladders Marker--Lane 2,3ú║cDNA--Lane 4ú║H 2 O--Lane 5ú║no RT.Tif]
